# Neuronal Modulation of Airway and Vascular Tone and Their Influence on Nonspecific Airways Responsiveness in Asthma

**DOI:** 10.1155/2012/108149

**Published:** 2012-10-23

**Authors:** Brendan J. Canning, Ariel Woo, Stuart B. Mazzone

**Affiliations:** ^1^Department of Allergy and Clinical Immunology, Johns Hopkins Asthma and Allergy Center, 5501 Hopkins Bayview Circle, Baltimore, MD 21224, USA; ^2^School of Biomedical Sciences, University of Queensland, Brisbane, QLD 4072, Australia

## Abstract

The autonomic nervous system provides both cholinergic and noncholinergic neural inputs to end organs within the airways, which includes the airway and vascular smooth muscle. Heightened responsiveness of the airways to bronchoconstrictive agents is a hallmark feature of reactive airways diseases. The mechanisms underpinning airways hyperreactivity still largely remain unresolved. In this paper we summarize the substantial body of evidence that implicates dysfunction of the autonomic nerves that innervate smooth muscle in the airways and associated vasculature as a prominent cause of airways hyperresponsiveness in asthma.

## 1. Introduction

With the exception of airway smooth muscle, perhaps no other group of cells has as clear a role in the pathogenesis of asthma as the neurons comprising the afferent and efferent innervation of the airways and lungs. The symptoms of asthma—wheezing, dyspnea, chest tightness, cough, reversible airways obstruction, mucus hypersecretion, and airways hyperresponsiveness—all inextricably link the nervous system to this disease. It is thus remarkable that in the 440 pages of the National Heart, Lung and Blood Institute (NHLBI) guidelines on asthma, nerves are mentioned in just one sentence [1]. Nerves are not mentioned at all in the British Thoracic Society (BTS) guidelines for asthma [2]. Even the recent and potentially landmark study by Peters et al. [3], in which the anticholinergic tiotropium was found to be at least as good as steroids or *β*-agonists (perhaps better) for treatment of asthma, nerves are not mentioned in the article itself nor in the accompanying editorial [4]. In this brief review we summarize the large body of evidence supporting a primary role for airway autonomic nerve dysfunction in the hyperresponsiveness of the airway smooth muscle in asthma. 

## 2. The Understated Role of Nerves in Asthma

Guidelines such as those produced by the NHLBI and BTS, in which immune cells including eosinophils are given a central role in asthma pathogenesis appropriately highlight the prominent feature of inflammation in the asthmatic lung. Inflammation may precipitate airways hyperresponsiveness [1, 5–8]. But the association between inflammation and airways hyperresponsiveness has probably been overemphasized [9–13]. The bias towards inflammation in asthma guidelines reveals the disproportionate influence immunologists, and allergists have had overdefining this disease for national and international medical organizations as well as their influence over the direction of asthma-related research. As asthma prevalence and asthma mortality rates have remained largely unchanged in the decades where inflammation has become a central theme in asthma research and therapy, it may be time for a new perspective on old concepts of the pathogenesis of reactive airways disease. 

Given the strong case for neural dysfunction in asthma, it is surprising how little attention airway nerves receive in the published literature. The under emphasis on nerves in asthma and the exaggerated influence of inflammation in asthma can be illustrated by comparing the scant references to neural mechanisms in this disease with the incessant discussions of eosinophils in asthma guidelines and in all of asthma-related literature. This is especially surprising, given how strong the evidence is in favor of neural mechanisms in asthma and how comparatively weak the evidence is supporting a role for eosinophils in this disease. Even the most ardent proponents might struggle to make a strong case for a role of eosinophils in asthma. There is no increase in the risk of asthma for patients with hypereosinophilic syndrome [14]. Nonasthmatic atopic patients develop a profound eosinophilia of the airways upon exposure to allergen but develop few if any of the symptoms of asthma [15, 16]. Many asthmatics have eosinophil levels in their airways and air spaces that are comparable to that of nonasthmatics [5, 6, 17]. Experimental therapies such as anti-IL-5 and recombinant IL-12 have a profound effect on circulating and airway eosinophil numbers and on allergen-induced recruitment of eosinophils to the airways but little or no effect on asthma symptoms in most patients and no effect on airways reactivity [18–21]. Even steroids, which markedly inhibit eosinophil function, survival and recruitment to the airways, have only modest effects on airways hyperresponsiveness [22]. And yet, in spite of what seems to be a clear role for the nervous system in asthma and at best a debatable role for eosinophils, nerves are mentioned in one sentence combined in the NHLBI and BTS guidelines while these same guidelines, cite eosinophils 85 and 43 times, respectively. This imbalance is pervasive in the published literature as well. Since 2001, several years after Leckie and colleagues reported their disappointing results with anti-IL-5, there have been over 4600 papers published with the keywords of “asthma” and “eosinophil” but less than 450 papers with the keywords “asthma” and “nerve”. Indeed, there have been more papers published with the keywords “eotaxin” and “asthma” over the past 10 years than papers with the keywords “asthma” or “COPD” and “nerves” combined.

The mechanisms of airways hyperresponsiveness are poorly understood. It seems all but certain that smooth muscle is central to regulating airways reactivity. However, studies of airways smooth muscle contractility *in vitro*, conducted using airways obtained from asthmatic and nonasthmatic lung donors, yield results that are somewhat varied [23–28]. Thus, an argument can be made that neither smooth muscle contractility (efficacy) nor responsiveness (potency) differs to any great extent between airways obtained from diseased or nondiseased patients. Bronchodilators are also largely equally effective in smooth muscle from asthmatics and nonasthmatics [29, 30]. The defect in asthma may therefore manifest only within the context of the intact body and lung.

 If, however, we accept the evidence in support of the hypothesis that airway smooth muscle accounts in large part for the most defining pathophysiological features of asthma (reversible airways obstruction and airways hyperresponsiveness) it is then critical to determine what ultimately regulates airway smooth muscle contraction. Airway smooth muscle generates little myogenic tone and so contraction depends upon the actions of contractile agonists. Despite an extensive list of autacoids and neurotransmitters that *can* contract human airway smooth muscle, a survey of the published literature suggests that only 3 endogenously released ligands, acetylcholine, histamine, and the cysteinyl-leukotrienes, reliably contract human airway smooth muscle to any significant extent and in physiologically relevant conditions in the airways of asthmatics. What so clearly defines the role of the nervous system in regulating the airways hyperresponsiveness in asthma is the indisputable source of the acetylcholine that regulates airway smooth muscle tone and the profound effects of anticholinergics on the airways obstruction and airways reactivity that define this disease.

## 3. Autonomic Innervation of Human ****Airway Smooth Muscle

The autonomic nervous system plays a primary role in regulating airway smooth muscle tone. The highly regulated activity of these nerves allows ongoing input to the airway smooth muscle such that basal tone is regulated on a breath by breath basis. The origin of this ongoing drive depends upon centrally (i.e., brainstem) mediated activity established by both respiratory and reflexive inputs [31–34]. In most animals and in humans, stimulation of airway autonomic nerves evokes near maximal constrictions of the airways through the actions of acetylcholine released from postganglionic parasympathetic nerves. Alternatively, activation of airway autonomic nerves can reverse completely spasmogen-evoked bronchoconstriction through the actions of noncholinergic neurotransmitters such as nitric oxide (NO) and vasoactive intestinal peptide (VIP) and related peptides. It follows logically that dysfunction or dysregulation of airway autonomic nerves is likely to contribute to the pathogenesis of asthma and COPD (reviewed in [35]).

**Figure 1:**
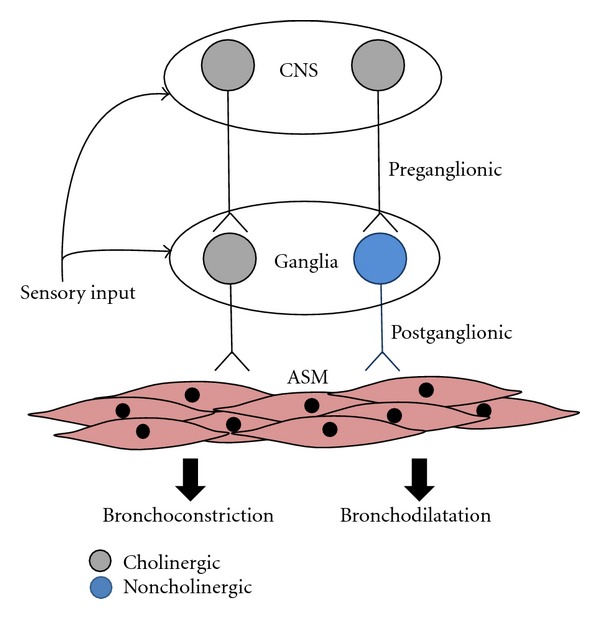
Two distinct vagal parasympathetic pathways regulate airway tone. Cholinergic preganglionic neurons originate in the brainstem and provide cholinergic drive to airway autonomic ganglia. Cholinergic postganglionic neurons are the major contractile input to the airways, whereas noncholinergic neurons expressing nitric oxide and vasoactive intestinal peptide provide the relaxant innervation to the airways. Airway sensory nerves contribute differential reflex regulation over cholinergic and non-cholinergic vagal pathways at the level of the brainstem and/or the airway ganglia. Dysfunction in ganglionic neurotransmission, neuromuscular transmission, or sensory reflexive control will precipitate changes in airway smooth muscle reactivity.

For years it had been widely assumed that noncholinergic neurotransmitters mediating relaxations of the airways were coreleased with acetylcholine from a single population of postganglionic parasympathetic nerves. It was further speculated that these noncholinergic cotransmitters served as a brake on the parasympathetic nervous system, preventing excessive constriction during periods of elevated autonomic tone. Our studies have revealed, however, that anatomically and physiologically distinct parasympathetic nerves mediate cholinergic contractions and noncholinergic (nitrergic) relaxations of the airways [36–38]. Importantly, reflexes differentially regulate these distinct parasympathetic pathways [34, 39]. The existence of two parasympathetic pathways with opposing actions on the bronchial musculature changes entirely how autonomic nerve-dependent regulation of airway caliber should be viewed. Bronchospasm could be evoked by increases in cholinergic nerve activity or withdrawal of nitrergic neural activity. Conversely, increased nitrergic nerve activity or decreased cholinergic tone could elicit bronchodilatation. The role of the autonomic nervous system in disease must also now be viewed differently. With distinct neuronal pathways mediating contractions and relaxations of airway smooth muscle, dysfunction or dysregulation of either parasympathetic pathway could account for the alterations in airway tone associated with asthma and COPD (Figure 1).

## 4. Autonomic Dysfunction and Asthma

 There is indisputable evidence supporting the hypothesis that dysregulation of airway cholinergic nerves contributes to the pathogenesis of airways obstruction and airways hyperresponsiveness. Cholinergic nerve-mediated obstruction of the airways is increased in asthma and COPD [28, 40]. Airways hyperresponsiveness is also associated with alterations in cholinergic nerve function. Anticholinergics markedly reduce (10–20-fold) or abolish airways reactivity to a wide variety of spasmogens and stimuli including prostanoids, histamine, bradykinin, capsaicin, hyperpnea, exercises and allergen (reviewed in [35]; Table 1). Airways hyperresponsiveness associated with extrapulmonary disorders may also be dependent upon alterations in airway autonomic control. Bronchospasm initiated by gastroesophageal reflux or airways obstruction associated with allergic rhinitis is prevented by anticholinergics. Similarly, in patients with upper respiratory tract infections, the marked increases in airways reactivity precipitated by the infection are reversed by atropine [41]. More recent studies suggest anticholinergics might be highly effective in treating asthma. Soon after the study of Peters et al. [3], which suggested that tiotropium was superior to steroids and *β*-agonists in controlling asthmatic airway function, 2 subsequent studies reported similar findings when using the ultra-potent and long acting anticholinergic [42, 43]. The reported effects of tiotropium on airway smooth muscle mass suggest that in addition to relieving functional obstruction, anticholinergics may play an important role in reversing airways remodeling [44].

Evidence suggesting that nitrergic parasympathetic nerves are dysfunctional in airways disease is circumstantial but compelling. In humans and in many animal species, adrenergic nerves are sparse or absent in the airways and without apparent influence over airway smooth muscle tone [45]. Consequently, nitrergic parasympathetic nerves represent the only functional relaxant innervation of airway smooth muscle. Importantly, in asthma, an inability to dilate with deep inspiration and not excessive smooth muscle constriction may underlie the pathogenesis of airways hyperresponsiveness [46]. In a preliminary report, inhibitors of NO synthase (NOS), which can inhibit relaxations mediated by airway nitrergic parasympathetic nerves, prevent the bronchoprotective effects of deep inspiration in normal patients [47]. Perhaps airway nitrergic nerves regulate airways reactivity by counteracting the actions of spasmogens through tonic, ongoing effects in the airways or by subserving a compensatory role with increased activity following challenge. Consistent with these hypotheses, NO synthase inhibitors exacerbate airways responsiveness to bradykinin in mild asthmatics, a compensatory mechanism that is lost in severe asthmatics [48]. Although the source of the nitric oxide was not determined in these clinical studies, experiments using animals and studies of human airway preparations indicate that parasympathetic nerves are one potential source [33, 49–52]. Pathological and molecular biological studies are also consistent with the hypothesis that dysregulation of airway noncholinergic nerves contributes to the pathogenesis of asthma and COPD. For example, arginase (which competes with neuronal NOS for the substrate L-arginine) activity is increased in models of asthma, thereby leading to a reduced capacity to produce neuronal NO [53]. Mutations in the gene encoding the neuronal isoform of NOS have been associated with asthma [54, 55]. These mutations are associated with a decrease in exhaled nitric oxide in asthma [56]. In fatal asthma, VIP-containing nerves have been reported to be sparse in the airways [57]. VIP and NOS are colocalized to airway ganglia [51]. All of these observations indicate that dysregulation of nitrergic parasympathetic nerves might contribute to the pathogenesis of airways diseases.

## 5. Autonomic Regulation of Vascular Tone ****in Asthma

**Figure 2:**
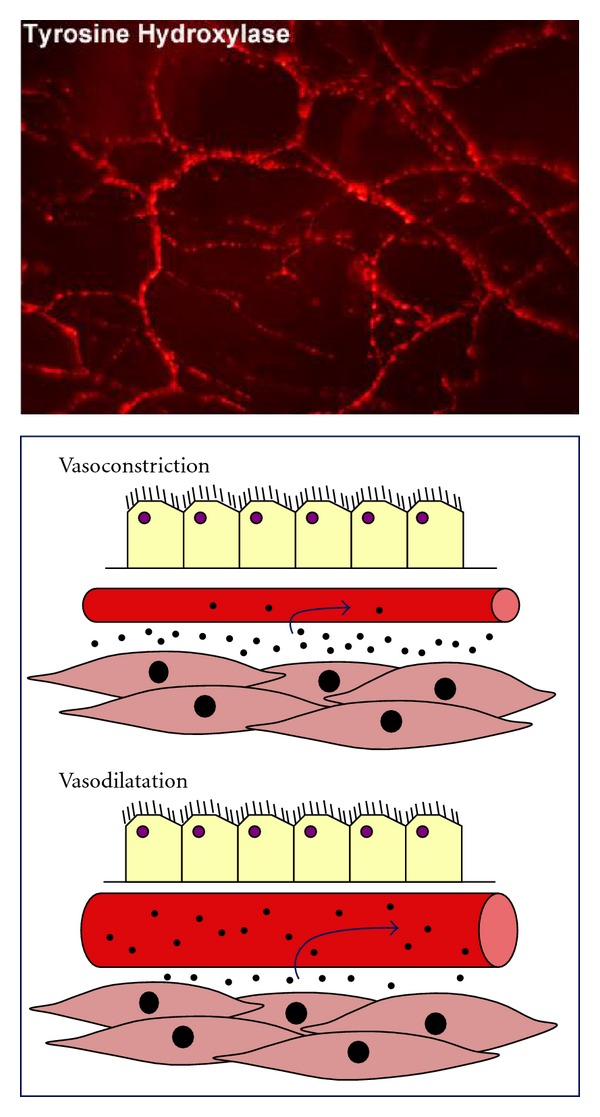
Airway vascular tone and blood flow regulate airway smooth muscle reactivity. The airway vasculature is densely innervated by sympathetic (tyrosine hydroxylase expressing) neurons which provide a basal level of adrenergic vascular tone. Soluble and insoluble particles in the airway wall are actively cleared by the submucosal vasculature. Increased blood flow is associated with increased clearance, and this can significantly modify airway smooth muscle reactivity to bronchoactive agents which are deposited onto, or generated within, the airway wall. See text for additional details.

Vascular beds in the airways play an important role in basal airway obstruction through the regulation of airway wall volume [58]. Mucosal edema is a prominent feature in the asthmatic airways, and this contributes significantly to airflow limitations [59]. The airway vasculature, however, can also directly modulate airway smooth muscle reactivity by regulating the clearance of bronchoactive agents from the airway wall. For example, animal studies have shown that vasoconstriction or reduced vascular perfusion of the airways significantly potentiates airway smooth muscle responsiveness to a variety of bronchospastic agents [60–62] (Figure 2). In asthmatics, intravenous angiotensin II increases methacholine bronchoconstriction but does not alter bronchoconstriction evoked by endothelin, an autacoid that constricts airway vascular smooth muscle (while methacholine relaxes vascular smooth muscle; [63, 64]). The loop diuretic furosemide also reduces airways reactivity to exogenous stimuli in humans [27], perhaps via a vasodilatory action since it does not relax airway smooth muscle *in vitro*. Similarly, *in vivo* epinephrine (a nonselective adrenergic agonist that would evoke both bronchodilatation and vasoconstriction) has no effect on reactivity yet *in vitro* (where the vasculature is no longer intact), it is more potent and efficacious than the *β*-adrenergic agonist albuterol at preventing airway smooth muscle constriction [65].

As is the case with airway smooth muscle, vascular smooth muscle possesses a baseline level of tone that is dependent upon ongoing activity of the autonomic nervous system [61]. However, unlike the airway smooth muscle, vascular tone is heavily dependent upon adrenergic sympathetic nerves acting via alpha-adrenergic receptors (reviewed in [66]). Neuropeptide Y also constricts the vascular smooth muscle secondary to sympathetic nerve activation, whereas activation of parasympathetic nerves evokes vasodilatation following the release of acetylcholine or nitric oxide and vasoactive intestinal peptide. In some species neuropeptide expressing sensory nerves can mediate vasodilatation via axon reflexes, although this is not likely a prominent mechanism of vasoregulation in humans.

## 6. Mechanisms of Autonomic Dysfunction

While it is clear that the nervous system is essential to the reactivity of the airways in asthma, it is unclear precisely what drives dysfunction of airway nerves in asthma. The simplest explanation might be that inflammation alters airway autonomic function in asthma. Airways inflammation has been associated with enhanced cholinergic responses following altered prejunctional control mechanisms (such as the muscarinic M2 autoreceptor that normally prevents acetylcholine release from nerves) or by sensitizing neurotransmission through the parasympathetic autonomic ganglia (the synaptic relay between pre- and postganglionic neurons) (reviewed in [35]). Nitrergic relaxant nerve responses may also be diminished in the asthmatic airways. The synthesis or degradation of the peptidergic and nitrergic neurotransmitters (or their substrates) utilized by nitrergic nerves may be perturbed in asthma via the actions of peptidases, free radical scavengers, and arginase. An alternative explanation is that the effects of airway inflammation are indirectly linked to autonomic nerve dysfunction. For example, sensitization and altered activity of airway sensory nerves is a common feature in asthma. Sensory nerves provide direct inputs to airway autonomic pathways, both at the level of the brainstem and the autonomic ganglia, and it is therefore likely that altered sensory function contributes to changes in autonomic drive to the airways [35].

## 7. Conclusions and Future Directions

It seems possible that an overemphasis on the role of inflammation in models of asthma, and less attention to the central role of airways hyperresponsiveness, has contributed to the frequent failure to translate promising therapeutic strategies discovered in animals into patients with asthma [67, 68]. Important insights into the mechanisms of inflammation in asthma have been established. In clinical studies, both leukotrienes and IL-5 induce pulmonary eosinophilia [69, 70], whereas in asthmatics, leukotriene modifiers and anti-IL-5 reduce eosinophil and basophil recruitment to the airways [19, 20, 28, 71]. The Th2 cytokines IL-4 and IL-13 also seem to play a role in asthmatic inflammation [1], but therapies targeting IL-5 [19–21], IL-4 [72], or IL-13 [73, 74] have provided little or no relief of asthma symptoms and little relief from airways hyperresponsiveness. By contrast, anticholinergics have proven remarkably effective at reducing the acute responses to allergen challenge and consistently decrease airways obstruction and airways reactivity in asthmatics. There is much unknown about the innervation of the airways. Given its central role in the pathogenesis of asthma, efforts to fill the many gaps in our understanding of airway neural control are warranted.

## Figures and Tables

**Table 1:** Effect of anticholinergics on airways hyperresponsiveness in asthma^a^.

Provocation	Effect
Beta blockers	Abolished response
Bradykinin	5-fold increase in PD_35_
Capsaicin	60% reduction in response
Distilled water	50–100% reduction in response
Exercise	30% reduction in response
Histamine	10-fold increase in PC_100_ SRaw
Hyperpnea	Abolished response in children
Prostaglandin D_2_	12- to 22-fold increase in PC_20_
Psychogenic stimulation	Abolished response
Reflux or esophageal acidification	Abolished response
Thromboxane A_2_	23-fold increase in PC_20_

^a^Anticholinergics used were either ipratropium bromide or atropine delivered via aerosol. Results reviewed in detail elsewhere [35]. Abbreviations: PC_20_ and PD_35_: provocative concentration (or dose) of agonist producing a 20% or 35% decrease, respectively, in forced expiratory volume in 1 sec (FEV_1_); PC_100_ SRaw: provocative concentration of agonist producing a 100% increase in specific airways resistance.
